# A Case of Plumb Stones Retained in the Intestines

**Published:** 1785

**Authors:** 


III. *A Cafe of Plumb Stones retained in the Inteftines. By* the fame.

TO the inftances of hardened fæces, occafioned by extraneous fubftances retained in the inteftines, collected by different writers †, I beg leave to add the following, as it

† See Philof. Tranfact. Vols. XXIII. and XXV. Edin. Med. Effays, Vols. I. and V. Effays, Phyfical and Literary, Vol. II.

may ferve to direct the attention of medical practitioners to a complaint which, though it has been very ably pointed out by the ingenious Mr. White *, of Manchefter, occurs, perhaps, more frequently than they are aware of.

Early in the year 1782, Mrs. Parker, wife of Mr. Robert Parker, Stone Mafon, at Greenbank, in Wyerfdale, about eight miles from Lancafter, applied to me on account of a painful affection of the bowels, which, fhe faid, had troubled her at intervals for more than feven years, and for which fhe had taken a variety of medicines without any benefit. The pain, fhe obferved, had been ufually moft fevere when fhe was either very loofe or very coftive.

I recommended different remedies to her, but with no better effect than thofe fhe had before taken; till, at length, in September, 1782, I gave her an active purge, which brought away a ball of hardened fæces that weighed three quarters of an ounce. Its nucleus was a plumb ftone.

The complaint did not terminate here; for the rectum was obftructed by another and much

* Cafes in Surgery, 8vo. 1770.

larger concretion, which ſhe was unable to void. To aſſiſt in its expulſion, I directed yolks of eggs, and oil, to be injected in the manner adviſed by Mr. White in ſimilar caſes; but this not proving ſufficient, I was obliged to introduce my finger into the anus, and having felt the concretion, eaſily extracted it. It weighed three ounces, and, like the other ſmaller one, had a plumb ſtone in its center.

The patient aſſured me that ſhe had never eat any plumbs for upwards of ſeven years; but ſhe recollected to have been firſt attacked with her complaint in the night after ſhe had eaten plumb tart, and ſaid ſhe was bled that night, and took ſeveral medicines, but never imagined that her complaint originated from the plumbs. She likewiſe obſerved, that ſhe had often felt two hard lumps under her ribs when the pain was ſevere.

After theſe concretions were brought away, her complaints ceaſed, and ſhe is now in perfect health.

*Lancaſter,*
Auguſt 18, 1785.

IV. *Caſe*

